# ZY3-02 Laser Altimeter Footprint Geolocation Prediction

**DOI:** 10.3390/s17102165

**Published:** 2017-09-21

**Authors:** Junfeng Xie, Xinming Tang, Fan Mo, Guoyuan Li, Guangbin Zhu, Zhenming Wang, Xingke Fu, Xiaoming Gao, Xianhui Dou

**Affiliations:** 1 Satellite Surveying and Mapping Application Center, NASG, Beijing 100048, China; xiejf@sasmac.cn (J.X.); txm@sasmac.cn (X.T.); ligy@sasmac.cn (G.L.); zhugb@sasmac.cn (G.Z.); wangzm@sasmac.cn (Z.W.); fxk@sasmac.cn (X.F.); gaoxm@sasmac.cn (X.G.); douxh@sasmac.cn (X.D.); 2 Key Laboratory of Satellite Surveying and Mapping Technology and Application, NASG, Beijing 100048, China; 3 School of Surveying and Geographical Science, Liaoning Technology University, Fuxin 123000, China; 4 School of Earth Science and Engineering, Hohai University, Nanjing 211100, China

**Keywords:** ZY3-02 laser altimeter, geometric calibration, footprint location prediction, rigorous geometric prediction model, pointing angle prediction, orbit prediction, attitude prediction

## Abstract

Successfully launched on 30 May 2016, ZY3-02 is the first Chinese surveying and mapping satellite equipped with a lightweight laser altimeter. Calibration is necessary before the laser altimeter becomes operational. Laser footprint location prediction is the first step in calibration that is based on ground infrared detectors, and it is difficult because the sample frequency of the ZY3-02 laser altimeter is 2 Hz, and the distance between two adjacent laser footprints is about 3.5 km. In this paper, we build an on-orbit rigorous geometric prediction model referenced to the rigorous geometric model of optical remote sensing satellites. The model includes three kinds of data that must be predicted: pointing angle, orbit parameters, and attitude angles. The proposed method is verified by a ZY3-02 laser altimeter on-orbit geometric calibration test. Five laser footprint prediction experiments are conducted based on the model, and the laser footprint prediction accuracy is better than 150 m on the ground. The effectiveness and accuracy of the on-orbit rigorous geometric prediction model are confirmed by the test results. The geolocation is predicted precisely by the proposed method, and this will give a reference to the geolocation prediction of future land laser detectors in other laser altimeter calibration test.

## 1. Introduction

Elevation measurement accuracy is a major evaluation element of surveying and mapping satellites, and elevation accuracy is a very important factor in surveying and mapping products, such as a digital orthographic model (DOM), digital elevation model (DEM), digital surface model (DSM), etc., [1,2,3]. A high-resolution remote sensing, surveying, and mapping satellite system can deliver high precise image products with precise auxiliary data, such as satellite attitude and orbit data. Surveying and mapping satellite always has a small base-to-height ratio less than 1, so elevation measuring accuracy will be a bottleneck in satellite photogrammetry [4,5]. In the past, we could apply ground control points (GCPs) or ground elevation control points to reduce elevation error of satellite image products when the covered area was small, but now we cannot apply this method to global surveying and mapping [6,7]. Thus, we should take some actions to improve elevation accuracy of satellite image products, and meet the demands of highly-precise global surveying and mapping.

A space-borne laser altimeter can obtain high-precision ground elevation information in theory, so we can take full advantage of its high precision, and implement it on the surveying and mapping satellite platform to improve the elevation accuracy of image products. Due to the influences of rocket thrust torque changes, platform vibration, external environment change, time inconsistency, atmospheric delay, and others, certain systematic errors are introduced to the pointing and ranging parameters of the laser altimeter, and these actual parameters are different with ones measured in the laboratory [8,9]. To eliminate the influence of system errors on the accuracy of the laser altimeter, it is necessary to carry out an on-orbit geometric calibration test. Several methods have been used to perform this test in past space-borne laser altimeter calibrations, and most can acquire reliable results, but on-orbit geometric calibration based on electro-optical ground based detector arrays is presently the best calibration method among them due to its reliability, operability, accuracy, etc., and this geometric calibration method is also one of the most important ways to calibrate the laser altimeter of an Earth observation satellite [10,11,12,13,14,15]. The electro-optical ground based detector array must be laid before the satellite passes the calibration area. Therefore, geolocation prediction of the laser footprint site is the first step in the calibration test. The prediction accuracy not only determines the size of the detector array layout, but it also influences the success or failure of the test.

The Geoscience Laser Altimeter System (GLAS), which is equipped on the Ice, Cloud, and Land Elevation Satellite (ICESat), calibration test was conducted by laying ground electro-optical infrared detectors, and applying an adjustment method to reduce the systematic errors based on GCPs derived from those “trigger on” detectors. The idea of calibration test is simple in concept, but there are some difficulties in the process, such as how to determine the size and geolocation of the electro-optical detector array. The laser emitting frequency of GLAS is 40 Hz, which can result in a spatial distance between along-track neighboring footprint centers where the distance is 172 m. We can capture no fewer than one footprint if the size of an along-track array is larger than 172 m and the cross-track is larger than 100 m [16,17,18,19].

Launched on 30 May 2016, by a Changzheng 4B rocket, ZY3-02 is the second satellite in the Ziyuan3 series. The first is ZY3-01, which was launched on 9 January 2012. ZY3-01 is equipped with high-resolution three-line CCD cameras and some other high-quality equipment. ZY3-01 has performed well in national stereo mapping missions, and the positioning accuracy of the product without GCPs reaches 10 m in China [20,21]. With this excellent performance, the ZY3-02 satellite almost follows ZY3-01, including systematic parameters, installation dependencies, and instruments. However, there are still some improvements in ZY3-02 compared to ZY3-01: the resolution of the multi-spectral camera is better, and ZY3-02 has an extra small experimental laser altimeter. The small laser altimeter, carried by ZY3-02, is designed to conduct a ground verification experiment to determine whether the altimeter can improve the elevation accuracy of surveying and mapping satellite image products [4]. Similarly, we should carry out the on-orbit geometric calibration test as the first step. Since the ZY3-02 satellite laser altimeter does not transmit back the waveform data, we can only choose the on-orbit geometric calibration method based on electro-optical ground-based detector arrays. Although applying the same calibration method, the calibration test is more difficult than GLAS, and the main reason is that the laser emitting frequency of ZY3-02 is much smaller than GLAS. The laser sample frequency of the ZY3-02 satellite is 2 Hz, so the spatial distance between the two neighboring footprints is about 3.5 km, which is calculated by the ZY3-02 satellite on-orbit flight speed. The large distance makes it impossible to use the traditional detector array layout method, like GLAS, whose detector array size is larger than the distance between the two neighboring footprints. To predict the geolocation of the ZY3-02 land laser detector array precisely, we should apply a new method that can predict the geolocation with a high accuracy. Since the accuracy of geolocation prediction determines the size of land laser detector array, the higher the precision of the geolocation prediction, the better for us that we can save resources and obtain high efficiency. However, considering the case and the accuracy that we require, the accuracy of geolocation prediction should be better than 300 m, which is our maximum limit.

Regarding the special requirements of the ZY3-02 satellite laser geometric calibration test, considering the analysis of the flutter and operational rules of the optical remote sensing satellite platform and the high-stability characteristic of the orbit and attitude, a laser footprint geolocation prediction model for laser altimeter geometry calibration based on an electro-optical ground based detector array is proposed, with the aim being able to determine the geolocation of electro-optical detector array in advance. The model includes three kinds of parameters that need to be predicted: pointing angle, orbit data, and attitude data [22]. As a small pointing angle error will be amplified on the ground, we apply terrain matching to obtain the prediction pointing angle with a pyramid strategy. Satellite centroid position determination accuracy is an important parameter in the laser altimeter land location process, and the satellite orbit determination accuracy has achieved a very high accuracy by GPS now (for the ZY3 series, the post-processed orbit determination accuracy is about 20 cm) [23,24]. We apply empirical acceleration to predict the orbit data based on highly-precise historical orbit determination data [25]. Attitude prediction data are also a necessary input parameter in the model, and we apply polynomial extrapolation, built by satellite platform attitude frequency analysis, to complete the attitude prediction based on highly-precise historical attitude determination data.

During the period of the ZY3-02 on-orbit test, we carried out five laser altimeter on-orbit geometric calibration tests during 7–26 August 2016 in Sonid Right Banner, Inner Mongolia Autonomous Region. The laser footprints predicted by the proposed model during the ZY3-02 satellite on-orbit test were all successfully captured, except for weather reasons, verifying the reliability of the prediction model. We conduct the comparison and analysis through many kinds of experiments, and determine comprehensive evaluations for the prediction model accuracy. The horizontal error is less than 150 m, which could meet the requirement of an actual outside calibration test.

The remainder of this paper is organized as follows: Following the Introduction, the fundamental theory of laser altimetry and fundamental information of the ZY3-02 laser altimeter are described in Section 2. Section 3 narrates the proposed model and its construction method, and different methods to get requisite three elements: pointing angle, orbit and attitude. In Section 4, we carry out different experiments to verify the geolocation prediction accuracy of the proposed model. Finally, the conclusions are given in Section 5.

## 2. Basic Information

### 2.1. Laser Altimetry

**Figure 1:**
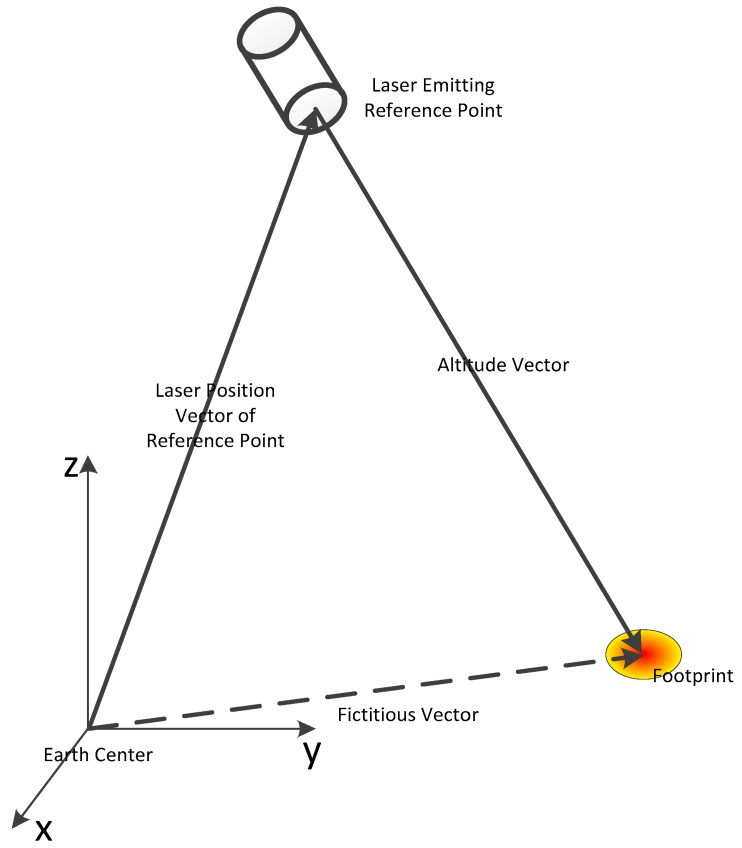
Altimetry concept.

Laser differs from other light in that it has: (a) a narrow spectrum; (b) high temporal coherence; and (c) high energy. A laser altimeter is an instrument that applies a laser to measure the land surface elevation; it can be equipped on an airplane or satellite platform. Since the laser altimeter applies a laser to measure the time interval between two points and calculates the distance between them with light speed, the instrument has a very high theoretical elevation measuring accuracy [18]. The laser altimetry concept can be showed as Figure 1.

There are some space-borne laser altimeters in different research and application fields that exhibit high accuracy and fine robustness. A laser pulse is a high-energy beam, and it can transmit with less energy attenuation and reflect more energy with a tolerable surface reflectivity if there are no occlusions. The GLAS aims to determine the long-term volumetric changes in the ice sheets at both the North and South Poles, and could provide specific information concerning variables such as land ice, cloud properties, atmospheric constituents, land cover aspects, and vegetation dynamics [16,26,27]. The primary objectives of the Mars Global Surveyor (MGS), a robotic spacecraft developed by NASA’s Jet Propulsion Laboratory, are to collect data about the Martian surface, atmosphere and magnetic properties, and to build a comprehensive dataset for future mission planning. The Mars Orbiter Laser Altimeter (MOLA) on the MGS aims to present an accurate mapping with an absolute accuracy of approximately 10 m vertically and 100 m horizontally [28,29]. The Near-Earth Asteroid Rendezvous (NEAR) spacecraft carried some instruments on a low-altitude orbit around an asteroid, and one of them is the NEAR Laser Rangefinder (NLR). The NLR provided sufficiently high resolution and accurate topographical profiles that, when combined with gravity estimates, and it would provide quantitative insight into the internal structure, rotational dynamics, and evolution of the asteroid [30,31]. The Lunar Orbiter Laser Altimeter (LOLA), an instrument in the payload of NASA’s Lunar Reconnaissance Orbiter (LRO) spacecraft, achieved success at its mission to measure the shape of the Moon by precisely measuring the range from the spacecraft to the lunar surface [32].

### 2.2. ZY3-02 Laser Altimeter

**Figure 2:**
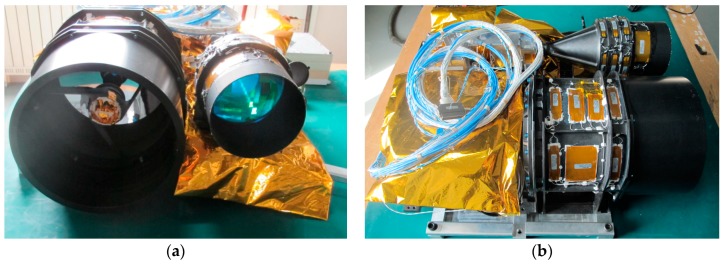
The spare one of the ZY3-02 laser altimeter: (**a**) the front of the laser altimeter; and (**b**) the top of the laser altimeter.

ZY3-02 is the first Chinese surveying and mapping satellite equipped with a small laser altimeter for elevation precision improvement of surveying and mapping [33]. The laser altimeter, equipped on a board, along with a multi-spectral camera, is an experimental payload firstly carried by a high-resolution stereo surveying and mapping satellite in China, and we aim to take it into space to test the fundamental performance of our domestic hardware. The outline of the ZY3-02 laser altimeter is shown in Figure 2, and it is a spare unit of the ZY3-02 laser altimeter. Its fundamental design parameters are shown in Table 1.

In the process of development and testing of a space-borne laser altimeter in the laboratory, its laser beam energy is concentrated and it is less affected by outside influences, hence, the pointing and ranging precision are very high (it reaches cm-level precision). However, the relative installation relationship between the payload and platform may change onboard due to platform vibration, environmental changes and other factors caused by the satellite launching, and this can cause horizontal and vertical errors [34]. In addition, the satellite sends a laser pulse to the ground from an orbit of about 500 km, and a delay occurs when the laser pulse passes through the atmosphere, which may cause a ranging error [35]. Thus, we should perform an onboard geometric calibration experiment on the ground to correct these errors.

There are several main methods of space-borne laser altimeter calibration: verification with high-resolution and precise airborne laser altimeter data and other elevation data [10]; systematic pointing error calibration with ocean and round-the-world scan crossovers using aerial photography to assist the space-borne laser altimeter calibration [17]; and high-precision onboard geometric calibration based on a land infrared detector array [11,18].

The method of land laser detector calibration, detecting energy emitted by an altimeter based on some electro-optical ground detectors and calibrating the altimeter ranging and pointing angle parameters by these detector locations, is reliable and easy to carry out. The detector is assembled with an infrared detection device, electrical circuit and outer shell. The detectors in the array should be triggered when illuminated by a laser pulse emitted by the altimeter. However, a necessary step in this method is the prediction of the laser location.

## 3. Methodology

### 3.1. Principle

Based on the principle of single-beam geometric positioning, we consider the satellite platform center of mass, the laser emission position, the GPS antenna centroid and the relative positional offset and rotational geometric relationship of the Earth ellipsoid surface, as well as the attitude changes of the satellite in the orbit coordinate system. The rigorous geometric prediction model is:

(1) $${\left(\begin{array}{c} X_{Pspot}\\ Y_{Pspot}\\ Z_{Pspot} \end{array}\right)}_{ITRF}=\;{\left(\begin{array}{c} {\tilde{X}}_{Ps}\\ {\tilde{Y}}_{Ps}\\ {\tilde{Z}}_{Ps} \end{array}\right)}_{ITRF}+R_{Orbit}^{ITRF}R_{BOD}^{Orbit}\left[\left(\begin{array}{c} \Delta X_{ref}\\ \Delta Y_{ref}\\ \Delta Z_{ref} \end{array}\right)+\tilde{\rho }\left(\begin{array}{c} \cos\left({\tilde{\beta }}_{P}\right)\cos\left({\tilde{\alpha }}_{P}\right)\\ \cos\left({\tilde{\beta }}_{P}\right)\sin\left({\tilde{\alpha }}_{P}\right)\\ \sin\left({\tilde{\beta }}_{P}\right) \end{array}\right)\right],$$

where ${\left(\begin{array}{ccc} X_{Pspot} & Y_{Pspot} & Z_{Pspot} \end{array}\right)}_{ITRF}^{T}$ is the predicted central position of the ground detector array layout in the Earth-fixed coordinate system; ${\left(\begin{array}{ccc} {\tilde{X}}_{Ps} & {\tilde{Y}}_{Ps} & {\tilde{Z}}_{Ps} \end{array}\right)}_{ITRF}^{T}$ is the predicted position of the satellite centroid in the earth-fixed coordinate system; $R_{Orbit}^{ITRF}$ is the rotation matrix transformed from the orbit coordinate system to the Earth-fixed coordinate system, both of which are related to the orbit position and velocity prediction results during the satellite over-flight; $R_{BOD}^{Orbit}$ is the rotation matrix transformed from the satellite body coordinate system to the orbit coordinate system, which is calculated by the predicted satellite body three-axis attitude roll, pitch, and yaw; ${\left(\begin{array}{ccc} \Delta X_{ref} & \Delta Y_{ref} & \Delta Z_{ref} \end{array}\right)}^{T}$ is the offset of the GPS phase center in the body coordinate system; $\tilde{\rho }$ is the estimated value of the range, which can be iteratively calculated from the ground DEM with predicted laser pointing angle, predicted orbit data and predicted attitude data, and the number of iteration calculation is always no more than 3; and ${\tilde{\alpha }}_{P}$ and ${\tilde{\beta }}_{P}$ are the predicted laser altimeter installment angles. We usually use the pointing ${\left(\begin{array}{ccc} \cos\left({\tilde{\beta }}_{P}\right)\cos\left({\tilde{\alpha }}_{P}\right) & \cos\left({\tilde{\beta }}_{P}\right)\sin\left({\tilde{\alpha }}_{P}\right) & \sin\left({\tilde{\beta }}_{P}\right) \end{array}\right)}^{T}$ as an input parameter.

The rigorous geometric prediction model includes three prediction parameters: pointing angle, orbit, and attitude, which will be predicted by corresponding method, respectively. The laser pointing prediction by terrain matching method and the optimum pointing angle value of onboard laser altimeter is estimated by pyramid iterative searching. The orbit prediction method based on empirical acceleration, combined with the playback data of precision ephemeris, is adopted. The method of frequency domain analysis, combined with the flutter characteristics of a satellite platform, is used to predict the attitude information of the over-flight satellite.

The rigorous geometric prediction model is the basis of the whole prediction method, and the laser altimeter pointing angle, attitude and orbit are also important for the prediction process. These data are the inputs of the model, so they will determinate the accuracy of the footprint site prediction. Now, we will introduce the basic theory of these parameter prediction methods in detail as follow:

(1) Laser pointing angle prediction

Due to the long distance between the satellite and the ground, a small laser pointing angle error will cause significant horizontal error. Taking the ZY3-02 satellite as an example, a pointing angle error of 1’ will cause about 150 m horizontal error on the ground at about a 506 km orbit. Therefore, it is necessary to predict the pointing angle of the laser altimeter firstly.

**Figure 3:**
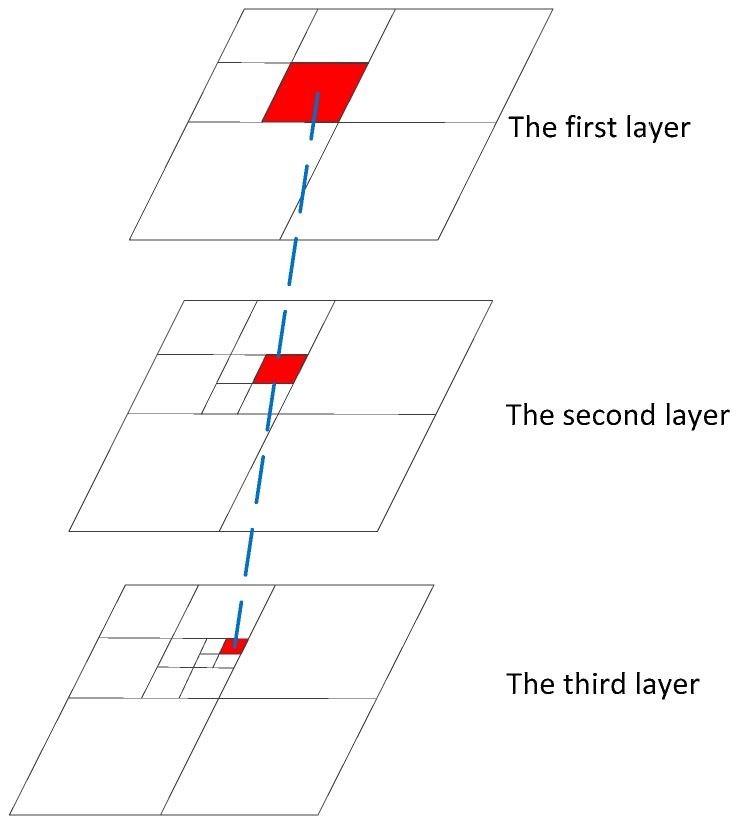
Pointing angle prediction with the pyramid strategy.

Putting different pointing angles, with certain orbit and attitude data, into the laser altimeter rigorous geometric model, will result in different elevation values [36]. Theoretically, the difference between the true elevation value and optimal calculation elevation value is the least among all values calculated by different pointing angles. In other words, after comparing the elevation value calculated by laser altimeter data with the true terrain value, the pointing angle corresponding to the smallest difference among sets of data is closest to the true one. We apply some groups of data (a group of data is a track of satellite data), including orbit, attitude, and pointing angle, to estimate the optimal pointing angle with least squares adjustment. To make sure that estimating high precise pointing angle, we always apply quite some (almost 1000) groups of data. As the calculation amount is so large, we apply the pyramid strategy to increase calculation efficiency. Based on this principle, a method of laser pointing prediction with pyramid terrain matching strategy is proposed. The calculation principle is shown in Figure 3.

By calculating the ground elevation information obtained by a space-borne laser altimeter of a corresponding whole track or even multiple tracks, the obtained elevation information is compared with the real terrain elevation value interpolated from DEM. When the elevation value is closest to the true terrain elevation value, the corresponding pointing angle is the optimal pointing angle. Due to the computational complexity, the pyramid method can be used to narrow the search area and obtain a higher accuracy of predicted laser pointing. The basic flow of this method is to traverse the optimal pointing angle from the first layer to the third layer, and the calculation scale is smaller and smaller.

The theoretical formula is:

(2) $$\left(\alpha_{r},\beta_{r}\right)=\overset{3}{\underset{i=1}{\Gamma }}\left(\overset{}{\underset{\alpha_{0}=\alpha_{i},\beta_{0}=\beta_{i}}{MIN}}\left(\overset{\begin{array}{l} m<p\left(i\right)/q\left(i\right)\\ n<p\left(i\right)/q\left(i\right) \end{array}}{\underset{m=0,n=0}{\mathbb{R}}}\left(E\left(\alpha_{i}=\alpha_{0}+m\times q\left(i\right)-p\left(i\right)/2,\beta_{i}=\beta_{0}+n\times q\left(i\right)-p\left(i\right)/2\right)\right)\right)\right),$$

where $\left(\alpha_{r},\beta_{r}\right)$ is the predicted pointing angle; $\overset{3}{\underset{i=1}{\Gamma }}$ represents the calculation from the first layer to the third layer among the pyramid; $\overset{}{\underset{\alpha_{0}=\alpha_{i},\beta_{0}=\beta_{i}}{MIN}}\left(\right)$ represents that $\alpha_{i}$, $\beta_{i}$ is the minimum value in the brackets, and we assign $\alpha_{i}$, $\beta_{i}$ to $\alpha_{0}$, $\beta_{0}$; q(i) is the step interval of layer i; p(i) is the size of the calculation region of layer i; $\overset{\begin{array}{l} m<p\left(i\right)/q\left(i\right)\\ n<p\left(i\right)/q\left(i\right) \end{array}}{\underset{m=0,n=0}{\mathbb{R}}}\left(\right)$ represents traversing the content in the bracket; m is the line number; n is the column number; and $E\left(\alpha_{i},\beta_{i}\right)$ is the elevation difference between the calculation and actual one in the pointing angle $\alpha_{i}$, $\beta_{i}$.

(2) Orbit prediction

The prediction accuracy of the satellite platform orbit position ${\left(\begin{array}{ccc} {\tilde{X}}_{Ps} & {\tilde{Y}}_{Ps} & {\tilde{Z}}_{Ps} \end{array}\right)}_{ITRF}^{T}$ is linearly related to the geolocation prediction accuracy of the laser altimeter footprint. A mature orbit prediction method based on empirical acceleration is adopted in this paper [37]. The method utilizes the original observation dual-band GPS data, which are transmitted in real-time to the ground and combined with the rapidly-released GPS orbit and clock correction products to calculate the precise orbit of the satellite for a certain time before its over-flight, and then predict the orbit data using the method of fitting the empirical acceleration.

The orbit prediction formula based on empirical acceleration is:

(3) $$\ddot{r}\left(t\right)=a\left(t,r,v,C_{D},C_{R}\right)+\xi_{i}\left(t\right)\left[a_{iR}e_{R}\left(t\right)+a_{iT}e_{T}\left(t\right)+a_{iN}e_{N}\left(t\right)\right],$$

where $\ddot{r}\left(t\right)$ is the second derivative of time in the conventional inertial system (CIS), the accelerated speed, and is the parameter needed to be calculated; t is a certain time; r is the position of satellite related to the time t; v is the velocity of satellite; $C_{D}$ is the resistance coefficient of atmosphere; $C_{R}$ is the light pressure coefficient of sun; a is the accelerated speed calculated by t, r, v, $C_{D}$, and $C_{R}$; $\xi_{i}\left(t\right)=\{\begin{array}{cc} 0, & t<t_{i}\\ 1, & t_{i}\leq t\leq t_{i+1}\\ 0, & t_{i+1}\leq t \end{array}$; $a_{iR}$ is the empirical acceleration of the i-th orbit, which acts on the radial direction; $a_{iT}$ is the empirical acceleration of i-th orbit, which acts on the tangential direction; $a_{iN}$ is the empirical acceleration of i-th orbit, which acts on the normal direction; $e_{R}\left(t\right)$ is the unit vector in the orbit radial direction; $e_{T}\left(t\right)$ is the unit vector in the orbit tangential direction; $e_{N}\left(t\right)$ is the unit vector in the orbit normal direction; $t_{i}$ is the beginning time of the i-th orbit; and $t_{i}$ is the beginning time of the (i+1)-th orbit, and also the end time of the i-th orbit.

The position r, velocity v, resistance coefficient of atmosphere $C_{D}$, and light pressure coefficient of sun $C_{R}$ of a certain time are derived from the fitting of precise historical orbit data. We can obtain the acceleration speed $\ddot{r}\left(t\right)$, which includes $\ddot{r}\left(t\right)=a_{0},a_{1},\cdots ,a_{n_{a}-1}$.

After that, we can obtain the $\left(P_{0},V_{0}\right)$, which are the position and velocity of the satellite that need to be predicted, by the following formula:

(4) $$\left(P_{0},V_{0}\right)={\left({P_{0}}^{\prime },{V_{0}}^{\prime },C_{D},C_{R},a_{0},a_{1},\cdots ,a_{n_{a}-1}\right)}^{T},$$

where ${P_{0}}^{\prime }$ and ${V_{0}}^{\prime }$ are the known initial position and velocity.

(3) Attitude prediction

The satellite body coordinate system should ideally coincide with the orbital coordinate system, but there is a certain angle difference between the satellite body coordinate system and the orbit coordinate system due to momentum wheel operation, solar pressure, satellite self-drift movement and other factors. Considering the high-stability characteristics of the ZY3-02 satellite platform, the attitude under the orbit system is generally smaller off-axis [38]. Given the existence of a series of fixed-frequency jitters of the satellite platform [8], an attitude prediction method based on frequency fitting is proposed in the paper with full utilization of the certain frequency.

Using the historical attitude data in a certain period before the satellite over-flight, with a series of predicted fixed frequencies of the satellite platform attitude jitter, generalized polynomials and trigonometric polynomials are introduced to extrapolate the cumulative attitude, which can ensure the satellite’s attitude precision. The attitude prediction formula based on frequency domain analysis is:

(5) $$f_{p}\left(t\right)=c_{0}+2\sum_{k=1}^{\infty }\left|c_{k}\right|\cos\left(2\pi kF_{}^{k}t+\theta_{k}\right),$$

where $f_{p}\left(t\right)$ is the predicted attitude, $c_{0}$ is a constant, k is the number of frequency bands, $c_{k}$ is the amplitude value of frequency band k, $F_{}^{k}$ is the frequency value, t is time and $\theta_{k}$ is the initial phase.

### 3.2. Workflow

**Figure 4:**
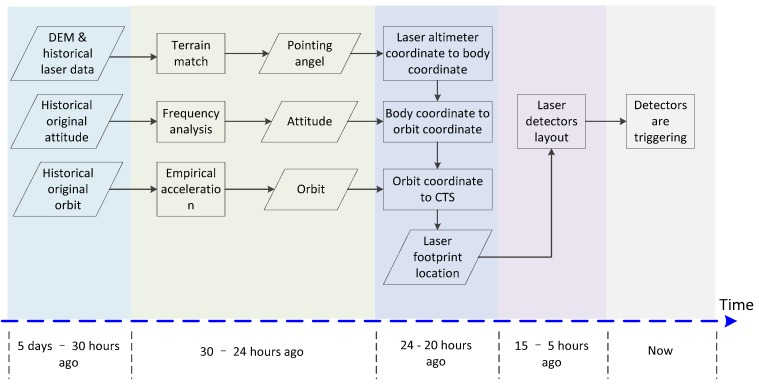
Workflow of laser altimeter footprint location prediction.

The workflow, i.e. the laser footprint prediction process, is shown in Figure 4. As in Figure 4, different operations should be finished at certain times, because we will obtain a more accurate prediction when applying the historical original attitude and original orbit closer to the moment of the test. The time of the historical data, to some extent, determines the overall prediction time. In addition, the time of the historical data also influences the accuracy of the prediction. We will finish the prediction test successfully if we adhere to the timetable, and the timetable reflects our experience after several onboard altimeter calibration tests.

The model is the core of the method, and it is related to some kinds of coordinate transformations. Now, we take the model as the technical basis to describe the workflow of the laser footprint prediction method.

(1) Laser altimeter coordinate to body coordinate

The installment angle between the laser altimeter and satellite platform is the rotation from the laser altimeter coordinate to the body coordinate, so the first step is obtaining the install angle. The pointing vector $\vec{P}$ can be calculated by the formula:

(6) $$\vec{P}={\left(\begin{array}{ccc} \cos\left(\beta \right)\cos\left(\alpha \right) & \cos\left(\beta \right)\sin\left(\alpha \right) & \sin\left(\beta \right) \end{array}\right)}^{T},$$

where α and β are the installment angles.

After the satellite is launched, the pointing angle is different from the one measured in the laboratory because the kinds of launching obstructions and the changing environment between the laboratory and space. The accuracy of the pointing angle will influence the geometric accuracy of the proposed prediction model. Thus, we should optimize the pointing accuracy to make it close to the true one by terrain matching with a pyramid strategy. This is a method of applying DEM or DSM and other elevation data as the reference to calculate the angle.

We set $\alpha_{0}$ and $\beta_{0}$ as the initial installment angles, set Δα and Δβ as the initial angle step sizes, and set $\alpha^{\prime }$ and $\beta^{\prime }$ as the traversal range. Firstly, we put $\alpha_{0}-\alpha^{\prime }+\Delta \alpha$ and $\beta_{0}-\beta^{\prime }+\Delta \beta$ into the laser altimeter’s rigorous geometry calculation model. The historical laser data usually include a mass of data that is processed by a rigorous geometric model, and then we can obtain the corresponding three-dimensional coordinate. The theoretical true elevation can be interpolated from the reference data based on the coordinate above, and we apply a bilinear interpolation as the interpolation method. The root-mean-square error (RMSE) between two groups of elevation, true elevation and calculated one, can be calculated. Then, we use $\alpha_{0}-\alpha^{\prime }+2\times \Delta \alpha$ and $\beta_{0}-\beta^{\prime }+2\times \Delta \beta$ to obtain a different RMSE, until applying $\alpha_{0}-\alpha^{\prime }+n\times \Delta \alpha$ and $\beta_{0}-\beta^{\prime }+m\times \Delta \beta$ to obtain the corresponding RMSE ($n=2\times \alpha^{\prime }/\Delta \alpha$, $m=2\times \Delta \beta /\beta^{\prime }$). We choose the α and β giving the smallest RMSE to be the prediction install angle. Then we put α and β into Equation (6) to obtain the prediction pointing vector $\vec{P}$.

(2) Body coordinate to orbit coordinate

There are two reasons why we select the orbit coordinate as the transition coordinate from the body coordination to a conventional terrestrial system (CTS). The first reason is that the angle changing between the body coordination and orbit coordination is small, because the satellite attitude control system maintains the balance in a timely manner to ensure the observation camera points toward Earth vertically. The second is that the satellite attitude in the orbit coordination system has a few certain constant frequencies (such as ZY3-02, which is about 0.7 Hz), and we can predict the attitude based on the frequency. The small attitude change amplitude limits the prediction error, and these constant frequencies offer the materials of prediction.

The ZY3 satellite has a frequency of about 0.7 Hz, which has been detected, and we can predict the attitude based on the frequency. We analyze the historical attitude data by FFT, and then we can obtain the main frequency of attitude f. We extrapolate the attitude as prediction attitude $f_{p}$ based on Equation (5). We can apply this method to predict the roll and pitch, but the yaw is invalid because there is almost a constant quantity difference in yaw, and the constant quantity could be calculated by historical attitude data easily. We define prediction attitude $f_{p}$ that includes roll, pitch and yaw. The $f_{p}$ can be divided into the three axis angles, roll, pitch, and yaw, and we can then calculate the rotation matrix from body coordinate to orbit coordinate by the following formula:

(7) $$R_{BOD}^{Orbit}=\left[\begin{array}{ccc} a_{1} & a_{2} & a_{3}\\ b_{1} & b_{2} & b_{3}\\ c_{1} & c_{2} & c_{3} \end{array}\right],$$

where $a_{1}\sim c_{3}$ can be indicated as follows:

$$\begin{array}{l} a_{1}=\cos\left(pitch\right)\times \cos\left(yaw\right)\\ a_{2}=-\cos\left(pitch\right)\times \sin\left(yaw\right)\\ a_{3}=\sin\left(pitch\right)\\ b_{1}=-\sin\left(roll\right)\times \sin\left(pitch\right)\times \cos\left(yaw\right)+\cos\left(roll\right)\times \sin\left(yaw\right)\\ b_{2}=\sin\left(roll\right)\times \sin\left(pitch\right)\times \sin\left(yaw\right)+\cos\left(roll\right)\times \cos\left(yaw\right)\\ b_{3}=\sin\left(roll\right)\times \cos\left(pitch\right)\\ c_{1}=-\cos\left(roll\right)\times \sin\left(pitch\right)\times \cos\left(yaw\right)-\sin\left(roll\right)\times \sin\left(yaw\right)\\ c_{2}=\cos\left(roll\right)\times \sin\left(pitch\right)\times \sin\left(yaw\right)-\sin\left(roll\right)\times \cos\left(yaw\right)\\ c_{3}=\cos\left(roll\right)\times \cos\left(pitch\right) \end{array}$$

(3) Orbit coordinate to CTS

Orbit prediction is a necessary step before laser footprint prediction, and the orbit prediction method is relative mature and widely used. We adopt the orbit prediction method based on empirical acceleration to predict the orbit, and the input data are historical original observation dual-band GPS data. We extrapolate the prediction orbit based on historical orbits.

The prediction orbit position is ${\left(\begin{array}{ccc} {\tilde{X}}_{Ps} & {\tilde{Y}}_{Ps} & {\tilde{Z}}_{Ps} \end{array}\right)}_{ITRF}^{T}$, and the prediction orbit velocity is ${\left(\begin{array}{ccc} {\tilde{V}}_{PX} & {\tilde{V}}_{PY} & {\tilde{V}}_{PZ} \end{array}\right)}^{T}$. The rotation matrix $R_{Orbit}^{ITRF}$ from the orbit coordinates to CTS can be calculated based on these prediction values, and the formula is:

(8) $$R_{Orbit}^{ITRF}=\left[\begin{array}{ccc} A_{1} & A_{2} & A_{3}\\ B_{1} & B_{2} & B_{3}\\ C_{1} & C_{2} & C_{3} \end{array}\right],$$

where $A_{1}\sim C_{3}$ can be determined as follows:

$$\begin{array}{l} \left[\begin{array}{c} A_{3}\\ B_{3}\\ C_{3} \end{array}\right]=\frac{{\left(\begin{array}{ccc} {\tilde{V}}_{PX} & {\tilde{V}}_{PY} & {\tilde{V}}_{PZ} \end{array}\right)}^{T}}{\left\|{\left(\begin{array}{ccc} {\tilde{V}}_{PX} & {\tilde{V}}_{PY} & {\tilde{V}}_{PZ} \end{array}\right)}^{T}\right\|}\\ \left[\begin{array}{c} A_{1}\\ B_{1}\\ C_{1} \end{array}\right]=\frac{{\left(\begin{array}{ccc} {\tilde{V}}_{PX} & {\tilde{V}}_{PY} & {\tilde{V}}_{PZ} \end{array}\right)}^{T}{}_{0}\times {\left[\begin{array}{ccc} A_{3} & B_{3} & C_{3} \end{array}\right]}^{T}}{\left\|{\left(\begin{array}{ccc} {\tilde{V}}_{PX} & {\tilde{V}}_{PY} & {\tilde{V}}_{PZ} \end{array}\right)}^{T}\times {\left[\begin{array}{ccc} A_{3} & B_{3} & C_{3} \end{array}\right]}^{T}\right\|}\\ \left[\begin{array}{c} A_{2}\\ B_{2}\\ C_{2} \end{array}\right]=\left[\begin{array}{c} A_{3}\\ B_{3}\\ C_{3} \end{array}\right]\times \left[\begin{array}{c} A_{1}\\ B_{1}\\ C_{1} \end{array}\right] \end{array}$$

(4) Obtaining the prediction location and deploying detectors

We can obtain the prediction laser footprint location in CTS based on the proposed prediction model, and it should be transformed to geographic coordinates. After the entire prediction process, there will be only 15 h when the satellite will pass the calibration area, so we set these detectors around the prediction location with a suitable layout in 10 h.

## 4. Experiments and Analysis

### 4.1. Theoretical Error Analysis

According to the rigorous geometric prediction model referred to in Equation (1), prediction errors are mainly caused by three factors: laser pointing angle prediction error, orbit prediction error, and attitude prediction error, as well as the ranging error caused by atmospheric delay and tide. In the following, we decompose each introduced error item and theoretically assess the prediction errors of the laser footprints in the along- and cross-track directions to obtain a final comprehensive theoretical prediction error.

**Figure 5:**
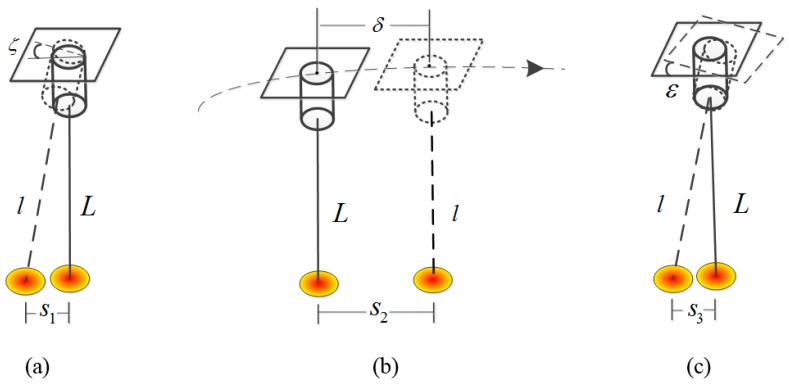
Decomposition of laser footprint prediction errors: (**a**) laser pointing errors; (**b**) orbit errors; and (**c**) attitude errors.

As shown in Figure 5, L represents the laser theoretical beam; l represents the real beam affected by these errors; (a) represents the ground positioning error $s_{1}$ caused by the laser pointing prediction error ζ on the ground; (b) represents the ground positioning error $s_{2}$ caused by the orbit prediction error δ; and (c) represents the ground positioning error $s_{3}$ caused by the attitude prediction error ε. The ground positioning errors $s_{1}$, $s_{2}$, and $s_{3}$ can be divided into the along-track direction error $s_{along}$ and cross-track direction error $s_{cross}$. The formula is:

(9) $$\{\begin{array}{c} s_{cross}=s\cdot \sin\left(\psi_{s}-\psi_{orbit}\right)\\ s_{along}=s\cdot \cos\left(\psi_{s}-\psi_{orbit}\right) \end{array},$$

where S is the ground error, $\psi_{s}$ is the azimuth of the ground errors and $\psi_{orbit}$ is the azimuth of the satellite track on the ground.

The ground positioning errors caused by the laser pointing prediction error, orbit prediction error and attitude prediction error can be decomposed into along- and cross-track directions according to the above formula. Each error can be divided into two components in the along- and cross-track directions. Since prediction error must be strictly limited in a tolerated range, we apply the maximum error as the analysis factor that ensures the prediction result is within control. Then, the integrated maximum errors in the along- and cross-track directions are calculated as:

(10) $$\{\begin{array}{c} S_{\max\_cross}=\left|s_{cross}^{\zeta }\right|+\left|s_{cross}^{\delta }\right|+\left|s_{cross}^{\varepsilon }\right|\\ S_{\max\_along}=\left|s_{along}^{\zeta }\right|+\left|s_{along}^{\delta }\right|+\left|s_{along}^{\varepsilon }\right| \end{array},$$

where $s_{cross}^{\zeta }$, $s_{cross}^{\delta }$, $s_{cross}^{\varepsilon }$ and $s_{along}^{\zeta }$, $s_{along}^{\delta }$, $s_{along}^{\varepsilon }$ are the error components in the along- and cross-track directions according to ζ, δ, and ε, respectively, calculated from Equation (9); and $S_{\max\_cross}$ and $S_{\max\_along}$ are the integrated maximum errors in the along- and cross-track directions, respectively.

We analyze these theoretical prediction errors from four sources: pointing, orbit, attitude, and others.

(1) The laser pointing prediction applies a terrain-matching method; the computation time is greatly reduced by the pyramid layer-by-layer calculation strategy, and the prediction precision of this method is relatively higher, but the prediction accuracy of laser pointing is limited by the referenced data accuracy. At present, the 30-m grid DEM is used to carry out the laser prediction pointing calculation; when the bottom pyramid traverse grid size is set to two seconds of arc, the resultant ground positioning error is about 5 m. Taking the maximum value of the two error sources, the laser pointing prediction error introduced in both the along- and cross-track directions is about 35 m.
(2) The orbit prediction based on empirical acceleration estimation requires the high-precision historical orbit data before the satellite passes the site. The accuracy of orbit determination can reach better than 20 cm by using the original real-time GPS data. On this basis, the empirical acceleration estimation method is adopted to predict the orbit, and good precision can theoretically be achieved. Before the on-orbit test, the predicted orbit is compared with the high-precision orbit several times, and the maximum error of the orbit prediction is about 150 m in the along-track direction and 20 m in the cross-track direction.
(3) As the ZY3-02 satellite adopts the large platform, to ensure the observation system is vertical to the ground, the momentum wheel is adjusting in real-time by the satellite attitude system; its three-axis design attitude stability is better than 5 × 10^−4^ °/s (3σ). Taking the fixed frequency of the platform along with processing into consideration, the accuracy of the attitude prediction method based on frequency domain analysis is better than 50 m in the along-track direction and 25 m in the cross-track direction.
(4) The errors caused by atmospheric delay and tide are mainly reflected in the ranging errors, and they have little effect on the horizontal prediction accuracy. Incorporating errors that may be introduced by other factors, the error is about 10 m.

In summary, the maximum theoretical prediction errors caused by the rigorous geometric prediction model are shown in Table 2.

As the design footprint size is 50 m, we set the size of laser detector array to be larger than 245 + 245 + 50 = 540 m and 90 + 90 + 50 = 230 m, and the laser footprint should be captured in theory. The maximal error is the extremity error of prediction, and we can refer the analysis result to design the style of the detector array.

### 4.2. Relative Accuracy Verification with Precise Post-Processed Data

We conducted two series of tests to show the relative prediction accuracy of the proposed method in this section. The first compares those prediction parameters with post-processed parameters to obtain the parameter prediction accuracy. The second compares the geolocation result calculated by the prediction parameters with that calculated by post-processed parameters to obtain the prediction parameter location accuracy.

The ZY3 image product could achieve the 1:50,000 mapping requirement without GCP, so the post-processed orbit and attitude data have very high accuracy. Based on this fact, we take the prediction footprint location, predicted with the calibrated pointing angle and the post-processed orbit and attitude parameters, as check data to verify the one predicted with the prediction point angle, orbit, and attitude data. We can verify these parameters’ accuracy to reflect the prediction accuracy, and analyze each kind of parameter to evaluate those parameter prediction methods. The first satellite calibration test was carried out on 9 August 2016. Next, we will take this test as an example to show prediction results in terms of laser pointing, orbit, and attitude. The basic information of five calibration tests will be introduced in Section 4.3 in detail, because the test in this section uses the post-processed data which cannot rely on the outside test. We apply the post-processed data to verify the relative accuracy of proposed prediction method in Section 4.2.

**Figure 6:**
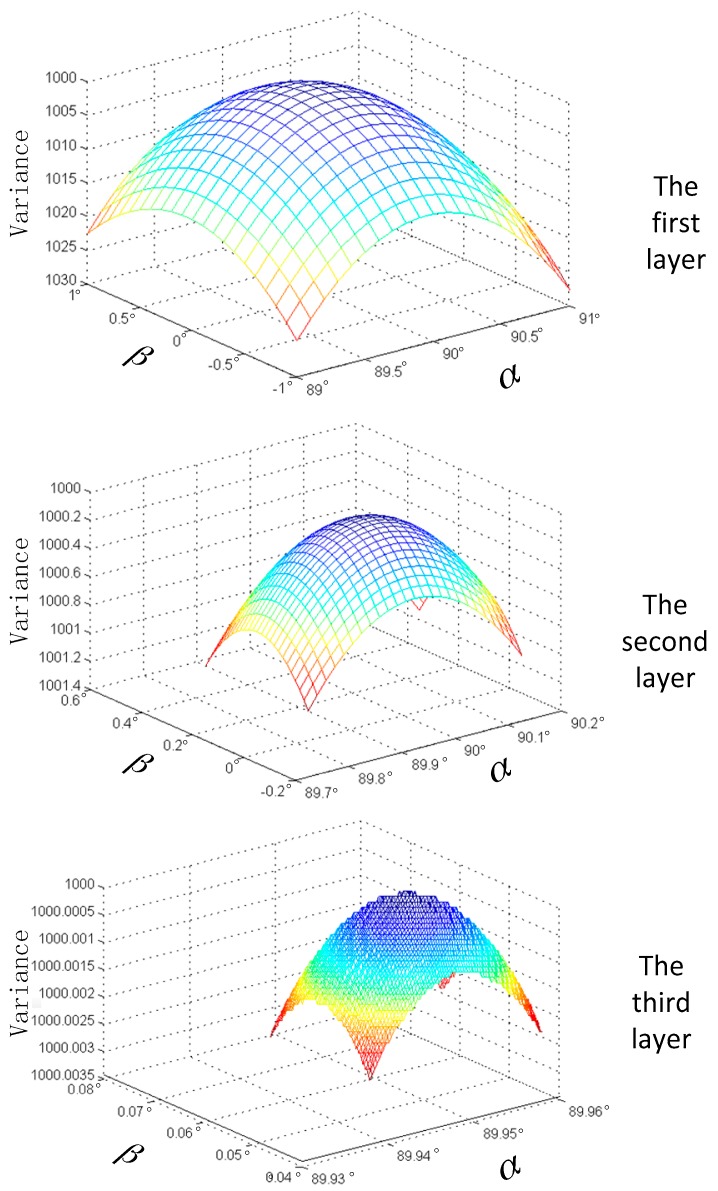
Pointing angle prediction.

As the pointing angle is predicted only once before the first onboard geometry calibration test, it can be improved by calibration after that. Therefore, we can compare the prediction pointing angle with the final one after all calibration tests. The prediction process of the pointing angle is shown in Figure 6. α and β are the pointing angle, and the z-axis is the variance of the elevation difference between the predicted and true elevation.

According to the method proposed in this paper, when conducting the initial laser pointing prediction, the traverse interval of the pyramid’s first, second, and third levels are 0.1°, 1′ and 1″, respectively. The search results of the three levels are shown in Figure 6. The AW3D30 data of the 30-m grid are adopted as the ground-referred terrain data [39,40]. The predicted results of laser pointing are α = 89.944456°, β = 0.046928°, and we set the original pointing angle as α = 90°, β = 0°. The final results of laser pointing are α = 89.949815°, β = 0.053393° after on-orbit geometry calibration, which correspondingly demonstrates higher prediction accuracy.

**Figure 7:**
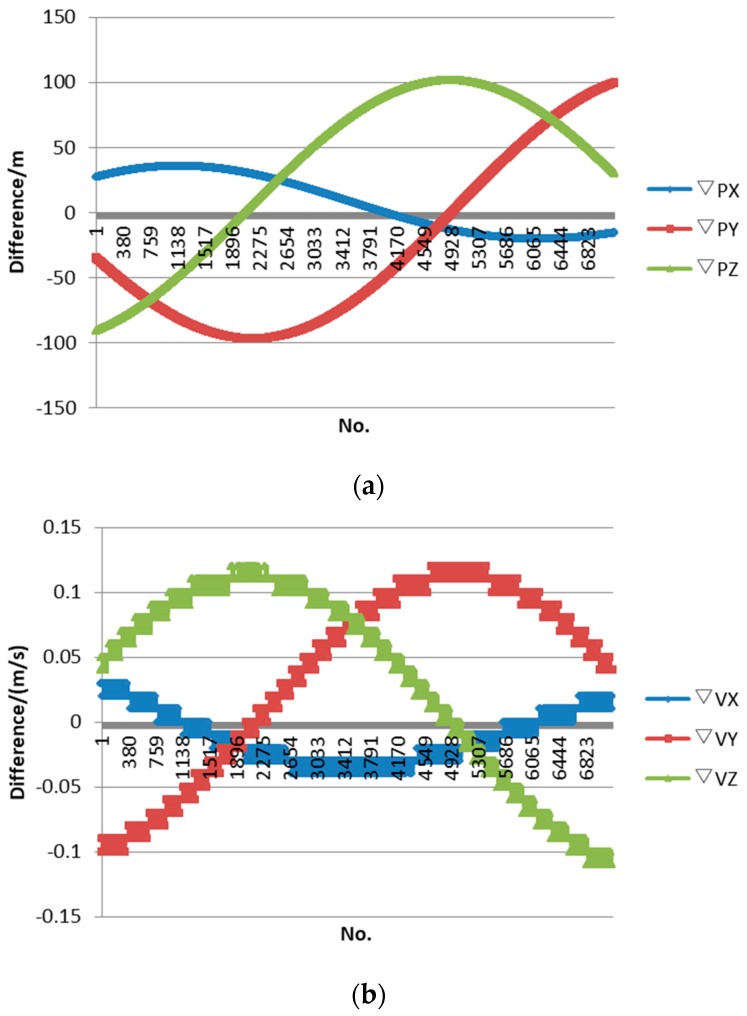
Difference between predicted ephemeris and true ephemeris: (**a**) position difference; and (**b**) velocity difference.

We adopted high-precision ephemeris data as the initial data, which were available 24 h prior to the test and had about a 40-h time span. We predicted satellite orbit parameters PX1, PY1, PZ1, VX1, VY1 and VZ1 before the on-orbit calibration test in advance. By adopting the original GPS data downloaded from the satellite when over-flying the calibration site, we had high-precision orbit parameters PX2, PY2, PZ2, VX2, VY2 and VZ2, which were processed through a post-precision orbit-determination algorithm. Through comparison, we could evaluate the accuracy of the predicted orbit. Figure 7 shows the difference between prediction ephemeris and real ephemeris for about one hour, including the calibration test period, with ordinals of orbit data on the horizontal axis while the position differences between the predicted orbit and the true orbit (the post-processing precision orbit parameter is better than 20 cm) on the vertical axis. The differences between parameters of predicted orbit and parameters of the high-precision orbit are ▽PX, ▽PY, ▽PZ, ▽VX, ▽VY and ▽VZ. The maximum position distance difference after orbit prediction is less than 100 m, and the maximum speed difference is within 0.15 m/s.

**Figure 8:**
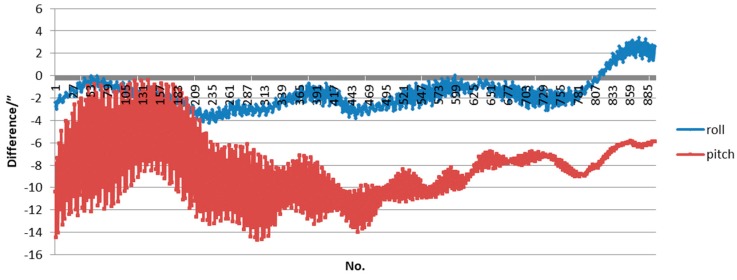
Difference between prediction attitude and true attitude.

Given the fixed frequency jitter of the satellite attitude platform, the prediction method of the attitude based on frequency fitting was adopted. As the influence of the yaw angle on the ground geolocation was very small and could be ignored, only the differences between the predicted roll angle and pitch angle and precision attitude data processed by a post-processed attitude determination algorithm were provided as follows. Figure 8 shows the difference of attitude data for 4.2 min, including the calibration test period, with ordinals of attitude data on the horizontal axis and angle difference on the vertical axis. As we can see, the difference of the roll angle is limited within 4″, while the pitch angle is limited within 15″.

**Figure 9:**
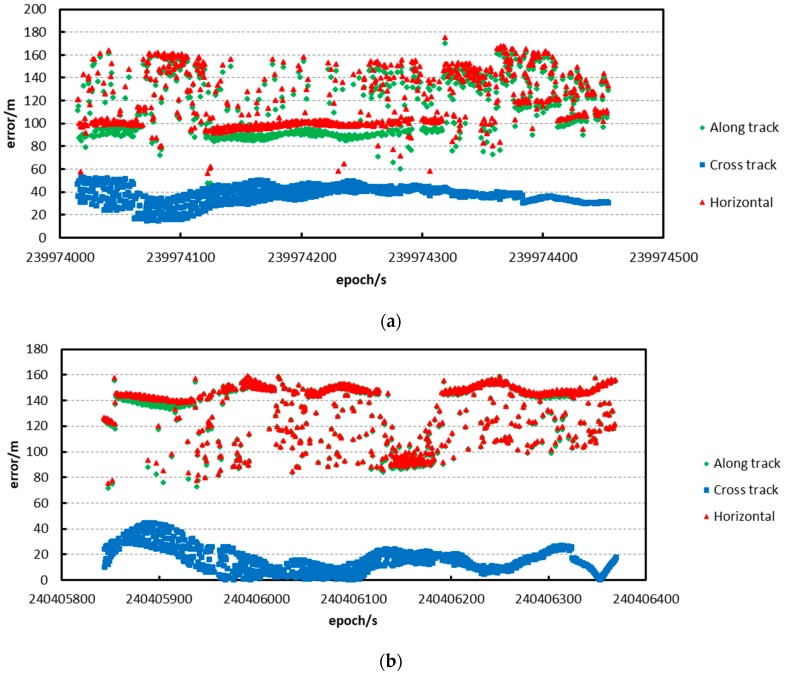

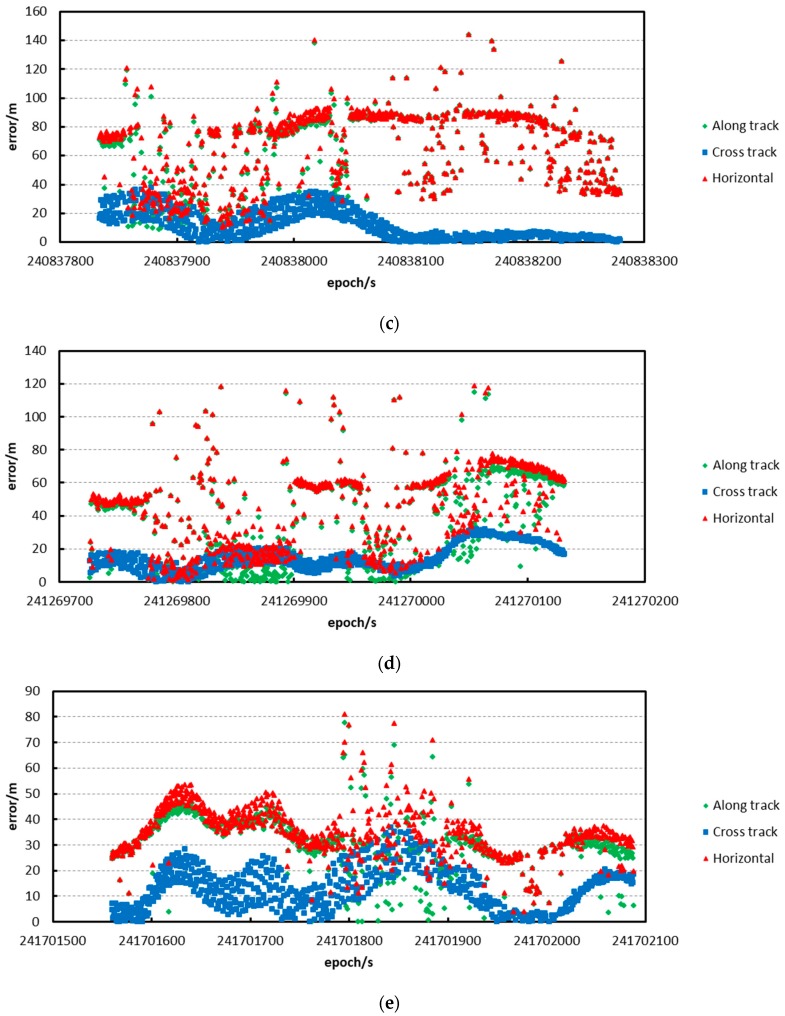
Prediction location error verified by the first method. The prediction error distribution of the test on: (**a**) 9 August; (**b**) 14 August; (**c**) 19 August; (**d**) 24 August; and (**e**) 29 August.

We then apply prediction pointing, attitude and orbit to calculate the footprint prediction location. Meanwhile, the pointing angle that is calibrated by the five tests, post-process attitude, and orbit, are applied to obtain the precise location. The differences between the predictions and the post-processed data in every test are shown in Figure 9.

From the resultant distribution figure, we find that the prediction error is no more than 200 m among all prediction points, visually. The prediction accuracy in the flat area is better than in rough terrain, and the prediction accuracy is relatively stable in flat terrain. The prediction accuracy is shown in Table 3 in detail.

In Table 3, we find that: (a) the along-track prediction accuracy is better than for cross-track, and the main error of the horizontal error is from the along-track prediction result; and (b) the prediction accuracy is better than 150 m.

### 4.3. Absolute Accuracy Verification with GCPs

**Figure 10:**
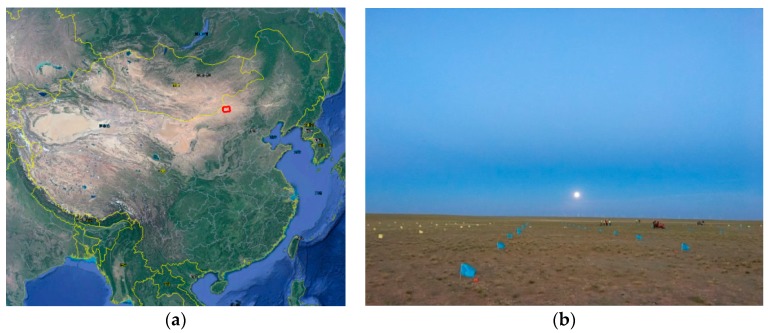
Laser altimeter calibration site: (**a**) calibration site location; and (**b**) detector arrays.

**Figure 11:**
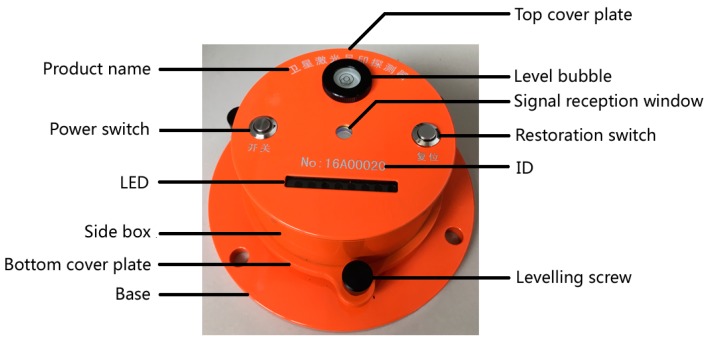
Laser detector.

By considering factors, including topography, climate, weather, transportation, vegetation, etc., and conducting an analysis, according to the five-day revisiting period of the ZY3-02 satellite, we decided to carry out ZY3-02 satellite laser altimeter on-orbit geometric calibration tests during 9–26 August 2016 (specifically, 9, 14, 19, 24 and 29 August) in Sonid Right Banner, Inner Mongolia Autonomous Region (111.550502°–112.765436°, 43.439333°–42.795053°), which is indicated in the red box in Figure 10a. As shown in Figure 10b, the test site terrain is flat and is dominated by pasture. During the testing period, it was sunny with little cloud cover. The ground laser infrared detector for the test is shown below in Figure 11.

According to the five-day revisiting period of the ZY3-02 satellite, five laser altimeter on-orbit calibration tests were conducted on 9, 14, 19, 24 and 29 August 2016. Before the over-flight of the satellite, previous accumulated data were utilized to predict laser pointing, orbit, and attitude. The results were put into the laser altimeter footprint-prediction model to predict the location, which provides the central geolocation reference for the deployment of ground detector arrays.

**Figure 12:**
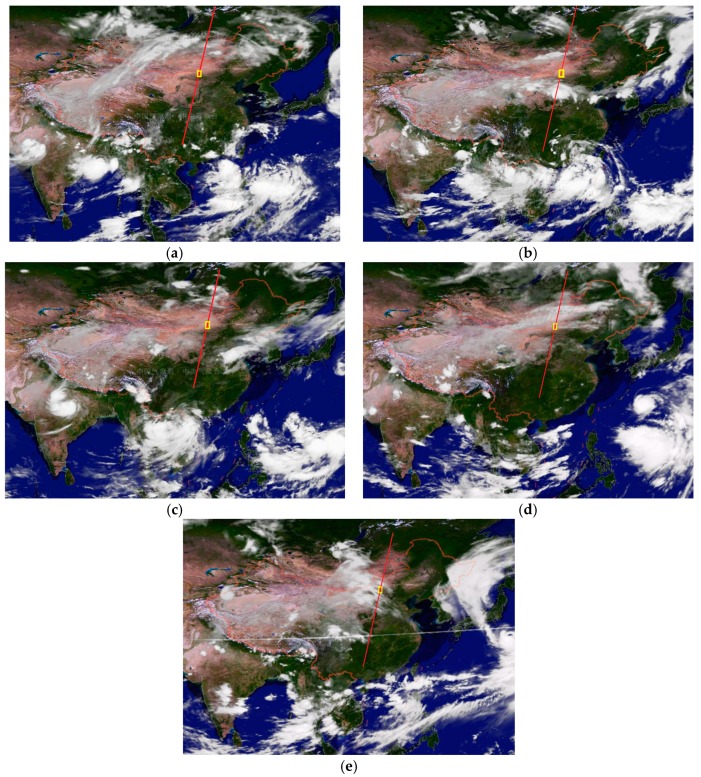
The five nephograms from the test period: (**a**) 9 August; (**b**) 14 August; (**c**) 19 August; (**d**) 24 August; and (**e**) 29 August. The red line is the satellite laser sub-point, and the yellow frame is the test alternative region.

The thickness of clouds will influence the test directly, and if it reaches a certain degree, the land infrared instruments will not detect the energy emitted by the altimeter or the laser acceptor cannot obtain valid data. We selected August as the test time. Although the cloud cover is relatively less than at other times in this period, there are some cases we do not want to encounter. The nephograms from five tests are shown in Figure 12.

**Figure 13:**
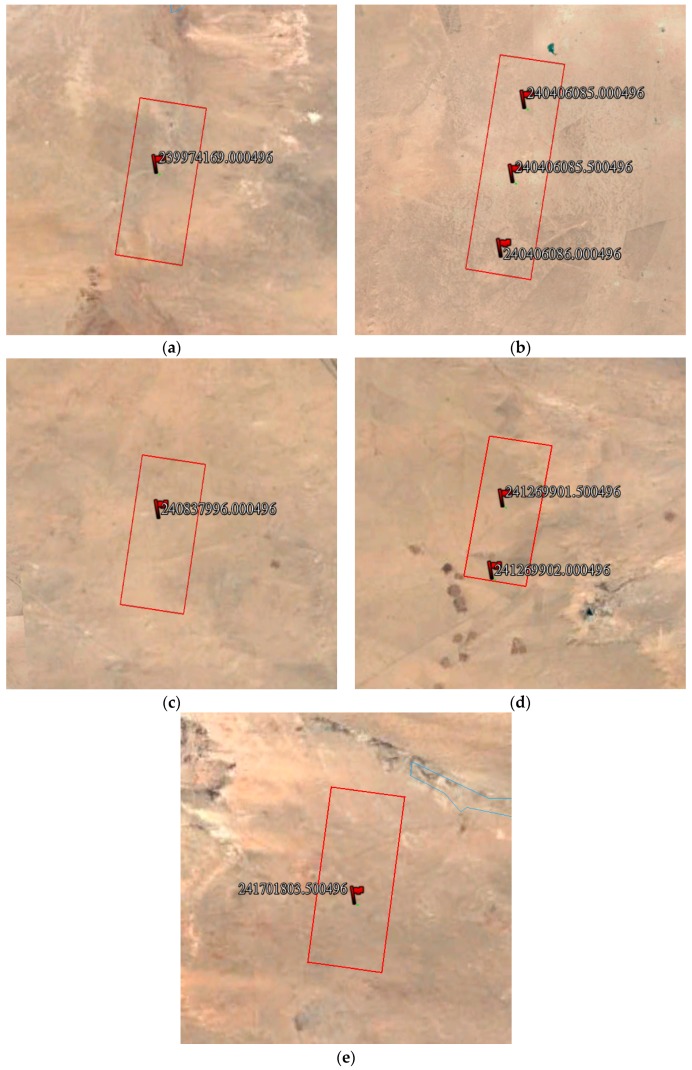
Laser footprint location prediction result; the location of small red flag is the prediction result, and the large red box is the alternative laser detector deploy area: (**a**) 9 August; (**b**) 14 August; (**c**) 19 August; (**d**) 24 August; and (**e**) 29 August.

Considering the basic conditions of laser detector layering, the temporary calibration site should be flat to decrease the influence of terrain, and the alternative region should avoid cropland and other special areas. In the alternative region, we applied the proposed method to predict the laser footprint location, and marked the location in Google Earth, as shown in Figure 13.

Before the over-flight of the satellite, previous accumulated data were utilized for predicting laser pointing, orbit, and attitude. The prediction results were put into the laser altimeter footprint prediction model to generate the center point coordinates of the laser footprint, which provides the geolocation reference for ground detector array deployment.

We took the prediction results shown in Figure 13 as the deployment center of laser detectors in the satellite on-board calibration test, and did five tests. The land laser detectors were triggered by the laser impulse, and that indicated the accuracy of the proposed prediction method could meet the need of outside calibration test. The tests on 9, 14 and 29 August achieved success. As the clouds thinned, the land detectors captured laser energy, but the on-board laser acceptor did not receive the return energy from the 14 August test. Due to clouds and other factors, the tests on 19 and 24 August failed. We list the results of all tests in Table 4.

Combined with the different responding energy levels of detectors in the ground footprint, the geodetic coordinates were measured by GPS-RTK. With the footprint central geolocation determination algorithm, we managed to precisely obtain the energy geometry center of the laser beam, which could be used as the true value of the laser footprint geolocation and compared with the predicted footprint geolocation to analyze the accuracy of the prediction model.

**Figure 14:**
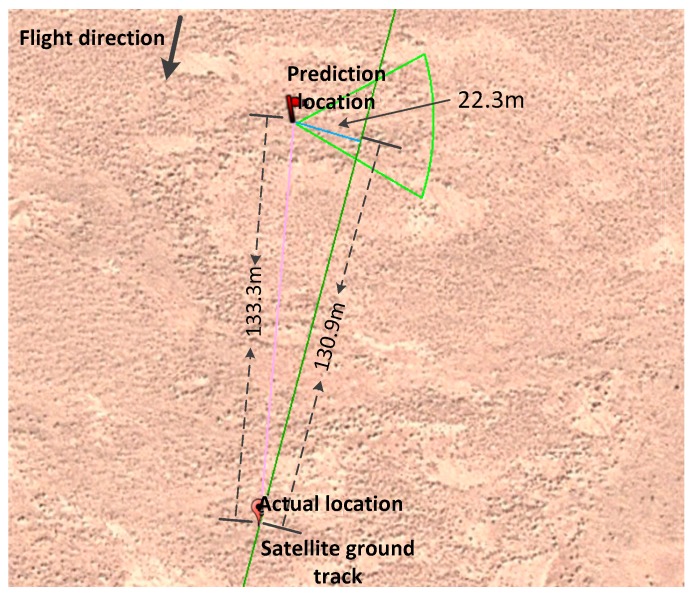
Error graph between the predicted location and actual value.

Taking the first calibration test on 9 August 2016 as an example, we determined the relationship between the predicted footprint geolocation and the actual location of the laser footprint captured by ground detector arrays, which is shown in Figure 14.

As indicated in Figure 14, the linear distance between the predicted and actual location is 133.3 m, with errors of 130.9 m and 22.3 m, respectively, when decomposed into along-track and cross-track directions. Since these errors were mainly caused by parameters of the prediction model, it is necessary to discuss separately the parameters of laser pointing, orbit, attitude and other factors, to further analyze each parameter’s error.

Prediction errors of laser pointing, orbit, attitude and other factors would all be reflected in the final prediction errors. To evaluate each error’s impact, we applied an independent analysis method. Taking orbit error analysis as an example, keeping other parameters constant, we used prediction orbit data and post-processing precision orbit data separately in the initial prediction model to calculate respective ground coordinates of the laser footprint. As precision orbit data had greater accuracy (less than 20 cm), the difference between the two coordinates was used as the orbit prediction error in the error analysis of orbit prediction.

**Figure 15:**
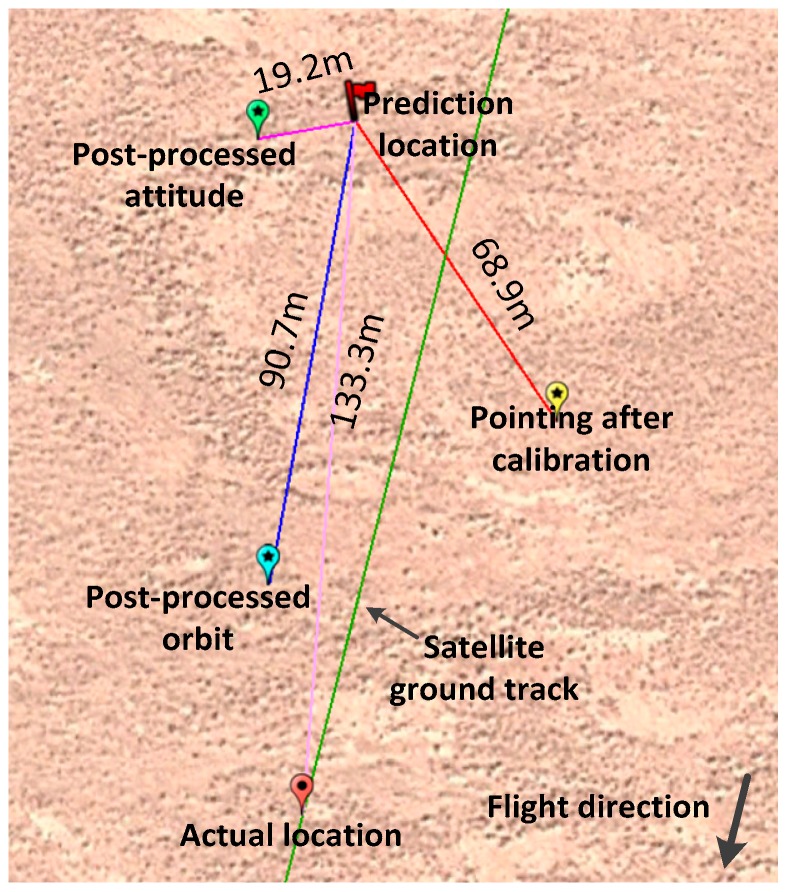
Decomposition of prediction errors.

As indicated in Figure 15, prediction location is a ground geolocation calculated by putting predicted laser pointing, predicted orbit and predicted attitude into the prediction model. Next, each error item will be analyzed through comparison.

Here, for the projection error, we set the “actual location” as the reference, and define the error direction to analyze them. The projection point on the north or west of the “actual location” is defined as “+”, and the one on the south or east is defined as “−”.

We will firstly analyze the impact of predicted laser pointing on geolocation prediction accuracy. After on-orbit geometric calibration, the elevation precision of altimeter data in the flat area is better than 1.0 m with calibrated laser altimeter pointing; therefore, the pointing accuracy is very high and can be used as a reference benchmark. By only putting the laser pointing after calibration into the prediction model and the orbit, attitude are still applying the predictions, we found that the geolocation varied in comparison with the predicted geolocation, which is indicated as “pointing after calibration” in Figure 15. The horizontal distance between “laser pointing after calibration” and “prediction location”, which is 68.9 m, was caused by pointing prediction error. When projected into the satellite’s flight direction, the error can be further decomposed into along-track error and cross-track error, which are 43.8 m and 53.2 m, respectively.

To analyze the orbit prediction error, we put the post-processing precision orbit data into the proposed laser geometric prediction model. The calculation result can be obtained, and it is indicated as the “post-processed orbit” in Figure 15. Through comparison with ground predicted geolocation, we determined the geolocation prediction error caused by the predicted orbit error, and there is a 90.7 m distance difference between the location calculated by the predicted orbit and the location calculated by post-processed precision orbit data. Then, projecting it onto the satellite’s flight direction, the error can be further decomposed into the along-track direction and cross-track direction errors, which are 90.4 m and −6.9 m, respectively.

The above method was also adopted for attitude error analysis, with post-processing precision attitude data as reference data. By putting post-processed precision attitude data into the prediction model, we can get the result shown in Figure 15, and we mark it as “post-processed attitude”. The distance between precision attitude determination and predicted geolocation is 19.2 m, while the along-track direction error and cross-track direction error are 7.4 m and −17.7 m, respectively.

For error analysis of the footprint geolocation prediction tests conducted on 14 and 29 August 2016, we adopted the same method, which we will not reiterate in this paper. All error items of the three prediction tests are shown below in Table 5.

According to Table 5, quantitative analysis of all error items of the laser altimeter rigorous geometry prediction model can be conducted. The first of the three tests above adopted a predicted laser pointing by the prediction method proposed in this paper. The other two tests adopted the pointing angle after the previous calibration process, so the pointing accuracy of the laser altimeter was improved. As we can see, the laser pointing angle accuracy is better than two seconds of arc after calibration, with random characteristics in the along- and cross-track directions. The orbit prediction error is the main error source of the prediction model, and it is random. Due to the features of a near-earth orbit (NEO) satellite, the geolocation error in the along-track direction is greater than in the cross-track direction, with a difference of one order of magnitude. We can see that the attitude error in the cross-track direction is larger than in the along-track direction, as the stability of the pitch angle is worse than that of the roll angle during ZY3-02 satellite on-orbit flight, which makes it difficult for the prediction attitude data to fit the attitude data with worse stability. Others in Table 5 mainly refer to the ground errors caused by factors such as atmosphere delay, tide, etc., which has lower error significance and is not systematic. As indicated in Table 5, the system error can be corrected by multiple calibrations; with the conducting of the calibration test and the promotion of laser pointing accuracy, the overall prediction error tended to decrease gradually. As the accumulated value of ground errors in along-track and cross-track directions caused by attitude, orbit, and pointing prediction are all greater than the overall ground errors, we can conclude that the various errors are random and there is a coupling relationship between them. To ensure the success of the calibration test, the ultimate detector array range depends on the maximum accumulated value of various prediction errors.

## 5. Conclusions

Prediction of laser footprint geolocation is a key step for on-orbit geometry calibration of a space-borne laser altimeter based on ground infrared detectors, and has a great influence for following tests to be conducted effectively. To complete on-orbit geometry calibration of the ZY3-02 laser altimeter, this paper builds a rigorous prediction model for the laser footprint geolocation prediction. Through prediction of laser pointing, orbit, and attitude, this model predicted the central geolocation of the laser footprint. By adopting this prediction method, five on-orbit geometry calibration tests of the ZY3-02 satellite laser altimeter were conducted at Suniteyou County, Inner Mongolia, in August 2016, where laser footprints were successfully captured. Taking the central geolocation of a laser spot captured in the site as a benchmark, we decomposed and analyzed various error items, and confirmed the effectiveness of the prediction model. Through quantitative comparison, the conclusions of the prediction model can be outlined as follows:

1. The prediction model built in this paper works fairly well at predicting the distribution geolocation of ground laser detector arrays for space-borne laser altimeter on-orbit geometry calibration, and the horizontal offset error is less than 150 m (prediction accuracy) in the final comprehensive prediction.
2. Through quantitative analysis of error items, the laser pointing prediction leads to ground geolocation error of about 70 m, which can be eliminated by post-processing of calibration and reaches about 3 m, less than 150 m of orbit prediction error, and less than 30 m of attitude prediction error.

Therefore, the rigorous laser footprint geolocation prediction model proposed in this paper can meet the need of on-orbit geometric calibration tests of space-borne laser altimeter, provide a reliable guarantee for successful implementation of ZY3-02 satellite laser altimeter on-orbit geometry calibrations based on ground detector arrays, and provide significant technical support for following on-orbit calibration of follow-up domestic low-frequency laser altimeters.

## Figures and Tables

**Table 1:** The fundamental design specification and characteristics of ZY3-02 laser altimeter.

Item	Value
Beam style	Single
Footprint size	50 m ^1^
Sample frequency	2 Hz
Attitude stability	5 × 10^−4^ °/s
Effective range	≥520 km ± 20 km
Detection probability	≥95%
Wavelength (Vacuum)	1064 nm
Pulse width	<7 ns
Range accuracy	<1.0 m (gradient <15°)

^1^ The value is recorded when the satellite platform is not swaying.

**Table 2:** Theoretic maximal error of rigorous geometric prediction model ^1^.

Error	Along-Track/m	Cross-Track/m
Laser Pointing	35	35
Orbit	150	20
Attitude	50	25
Other	10	10
Total	245	90

^1^ we do not define the direction of error, and only care about the maximal error.

**Table 3:** Prediction location accuracy condition verified by the post-processed data.

Test time	The No. of Laser Point	Along track		Cross track		Horizontal	
		Average	RMSE	Average	RMSE	Average	RMSE
9 August 2016	811	112.3	25.8	36.9	7.2	118.8	23.9
14 August 2016	903	131.2	21.3	15.5	9.6	132.5	21.1
19 August 2016	762	63.5	26.3	11.8	9.9	65.8	25.1
24 August 2016	718	39.5	26.0	14.6	7.5	43.6	24.9
29 August 2016	715	31.3	10.3	13	8.1	35.0	9.7

**Table 4:** Preview of altimeter calibration tests.

Time	Weather	Arrangement	Deploy Distance	Spot Shape
Second 29, 11:29 a.m., 9 August 2016	Excellent air quality, Spacing breeze 2–3 level, no clouds, high visibility.	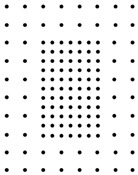	20 m	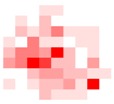
			40 m	
Seconds 5, 5.5, and 6, 11:28 a.m., 14 August 2016	Excellent air quality, Spacing breeze 3 level, slight clouds, relatively high visibility.	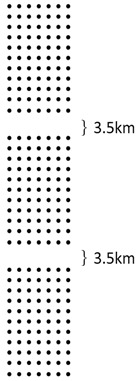	25 m	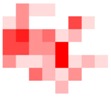
				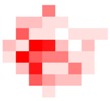
				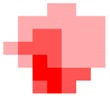
Second 36.5, 11:26 a.m., 19 August 2016	Normal air quality, Spacing breeze 3 level, cloudy, ordinary visibility.	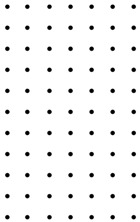	20 m	Rainy, thick cloud which could not be penetrated by laser
Seconds 1.5 and 2t, 11:25 a.m., 24 August 2016	Normal air quality, Spacing breeze 3 level, cloudy, ordinary visibility.	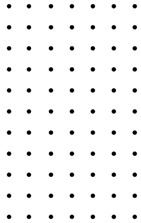	20 m	Rainy, thick cloud which could not be penetrated by laser
Second 23.5, 11:23 a.m., 29 August 2016	Excellent air quality, Spacing breeze 3 level, lightly cloudy, ordinary visibility	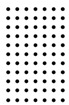	10 m	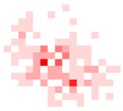

**Table 5:** Accuracy analyses of the prediction model.

Time	Error Direction	Ground Geolocation Error Caused by Each Error/m				
		Pointing	Orbit	Attitude	Others	Overall
Second 29, 11:29 a.m., 9 August 2016	Horizontal	68.9	90.7	19.2	12.4	133.3
	Along-track	43.8	90.4	7.4	−10.7	130.9
	Cross-track	53.2	−6.9	−17.7	−6.3	22.3
Second 5, 11:28 a.m., 14 August 2016	Horizontal	21.6	149.8	18.6	11.4	126.9
	Along-track	18.0	−149.4	8.2	5.7	−117.5
	Cross-track	11.9	7.1	16.7	2.9	38.6
Second 23.5, 11:23 a.m., 29 August 2016	Horizontal	2.6	27.3	20.4	19.0	43.5
	Along-track	1.8	−27.2	3.5	0.8	−21.1
	Cross-track	1.9	0.9	20.1	15.2	38.1
